# A novel method of transapical ProtekDuo rapid deployment cannula as temporary left ventricular assist device

**DOI:** 10.1093/jscr/rjad246

**Published:** 2023-06-28

**Authors:** Harish Ravipati, Nishank P Nooli, Charles W Hoopes, Enrique Gongora, Britt McILwain, Charles M Tyndal, Erik J Orozco-Hernandez

**Affiliations:** Department of Medicine, Division of Cardiovascular Disease, The University of Alabama at Birmingham, Birmingham, AL, USA; Department of Anesthesiology, The University of Alabama at Birmingham, Birmingham, AL, USA; Department of Surgery, Division of Cardiothoracic Surgery, The University of Alabama at Birmingham, Birmingham, AL, USA; Department of Surgery, Division of Cardiothoracic Surgery, The University of Alabama at Birmingham, Birmingham, AL, USA; Department of Surgery, Division of Cardiothoracic Surgery, The University of Alabama at Birmingham, Birmingham, AL, USA; Department of Surgery, Division of Cardiothoracic Surgery, The University of Alabama at Birmingham, Birmingham, AL, USA; Department of Surgery, Division of Cardiothoracic Surgery, The University of Alabama at Birmingham, Birmingham, AL, USA

**Keywords:** Cardiogenic shock, mechanical circulatory support, transapical ProtekDuo

## Abstract

Peripheral extracorporeal membrane oxygenation (ECMO) is one of the most common strategies for cardiogenic shock. ECMO cannulation is associated with an increased risk of complications. We describe a minimally invasive, off-pump technique to provide adequate hemodynamic support and left ventricular unloading. A 54-year-old male with nonischemic cardiomyopathy and severe peripheral vascular disease with cardiogenic shock was initially supported with inotropes and an intra-aortic balloon pump. Despite continued support, he continued to deteriorate, and we escalated to a temporary left ventricular support with a CentriMag, using a transapical ProtekDuo Rapid Deployment cannula via mini left-thoracotomy. This approach provides adequate hemodynamic support, left ventricular unloading and early ambulation. After 9 days, the patient’s functional status was improved and was medically optimized. The patient received a left ventricular assist device as destination therapy. He was discharged home, resumed his normal activities and has been doing well for more than 27 months.

## INTRODUCTION

Temporary mechanical circulatory support (MCS) utilization has expanded over the past decade in patients with cardiogenic shock and progressive end-organ injury [1]. Peripheral extracorporeal membrane oxygenation (ECMO), central ECMO and axillary Impella 5.5 are the common MCS that include significant invasive approach and the need for possible cardiopulmonary bypass [2]. We present a temporary left ventricular assist device (LVAD) using a transapical dual lumen cannula with novel technical modifications that improve postoperative management and safety [3–5].

## CASE REPORT

A 54-year-old male presented with acute exacerbation of nonischemic cardiomyopathy. The patient was transferred with IABP. Right heart catheterization and blood work revealed an RAP 13 mmHg, mean PAP 32 mmHg, PCWP 23 mmHg, Fick CI 1.94 L/min/m^2^, Ck 1.7 mg/dl and total bilirubin 2.6 mg/dl. Chest X-ray demonstrated bilateral pulmonary edema. TTE showed an LVEF < 20%, and RV function was severely reduced. The patient was not considered for a heart transplant based on his smoking history and severe PVD. He was not a candidate for femoral ECMO or Impella, given his vascular disease. Impella 5.5 was not available at that time. A multidisciplinary team determined that the best approach would be a transapical ProtekDuo RD as a bridge to LVAD.

**Figure 1:**
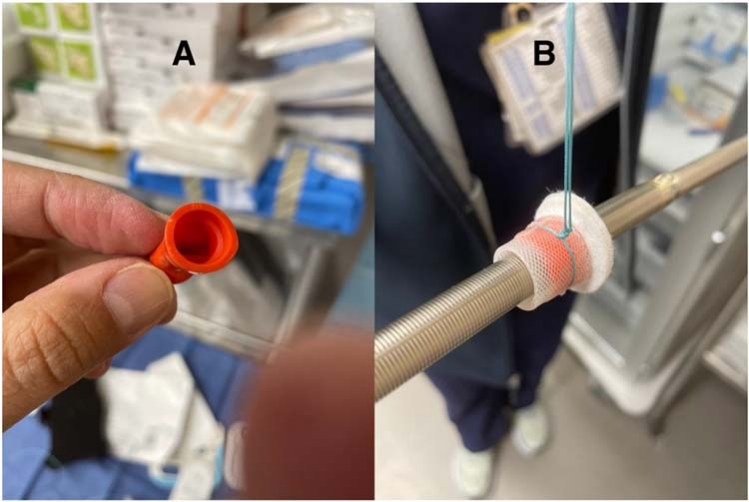
(**A**) Sterile hemostatic red cap from a 21 Fr Edwards Thruport EndoReturn arterial cannula; (**B**) Ethibond suture

**Figure 2:**
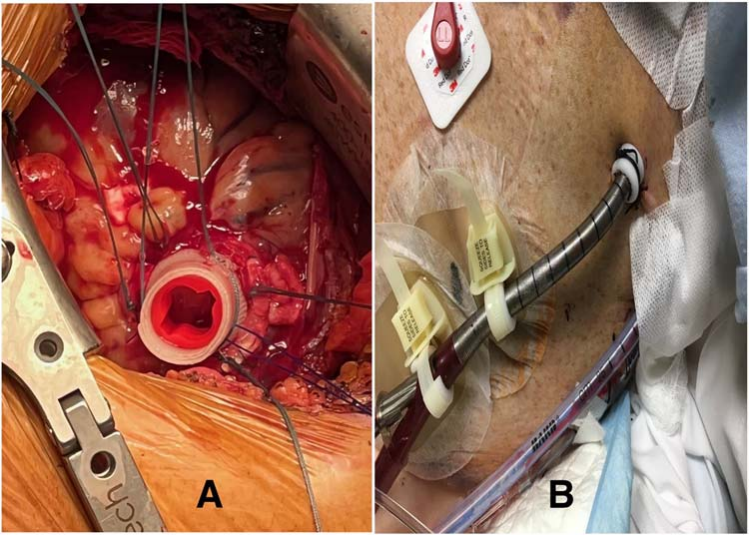
(**A**) Modified CentriMag™ apical sewing ring; (**B**) apical access of ProtekDuo RD with Hollister devices

The difference in the diameter of the ProtekDuo RD^®^ (31 Fr) and the internal diameter of the CentriMag™ 34Fr apical ring poses the risk of poor fixation and bleeding. To overcome this challenge, we placed a plastic washer (sterile cap from a 21 Fr Edwards arterial cannula) inside the sewing ring (Fig. 1). A small left anterior thoracotomy was performed, and the apex of the heart was identified. A modified CentriMag™ apical sewing ring was anastomosed to the apex with four full quadrant thickness 3-0 pledgeted Ethibond^®^ sutures and a 3-0 polypropylene running suture. Under TEE guidance, the apical myocardium inside the ring was accessed with a needle. Using the Seldinger technique, a 0.035″ Glidewire^®^ (Terumo) was placed into the left ventricle and passed antegrade through the aortic valve into the ascending aorta. A 32F chest tube was placed inferior to the thoracotomy, making a tunnel. The Glidewire^®^ was exteriorized through the chest tube. The Glidewire^®^ was exchanged for an 0.035″ Amplatz Super Stiff™ wire. The chest tube was removed. A 31Fr ProtekDuo RD^®^ cannula was lubricated and advanced over the wire. Once the cannula was placed through the modified ring, a # 2 Ethibond^®^ suture was tied around, securing together the cannula (Fig. 2). The double-lumen cannula was connected to a paracorporeal CentriMag™ pump, and there was immediate LV decompression and decreased PCWP. The patient was able to ambulate within 48 h of the procedure and was subsequently implanted with a durable LVAD after 9 days. The cannula was free of any thrombus at the time of explantation and has been doing well for more than 27 months.

## DISCUSSION

PROTEK Duo-RD cannula is primarily designed for VV ECMO. Khalpey *et al*. [1] used a modified PROTEK Duo cannula by cutting it shorter at the distal end; this modification is not recommended due to the increased risk of injury to the ventricle, aortic valve or aorta. Avery *et al*. [2] did not modify the length but found important challenges as the cannula length and design were not appropriate. There are a few reports of using PROTEK Duo-RD cannula. Lima *et al*. [3] described a case successfully bridged to recovery. Belani *et al*. [4] reported a patient supported for 3 weeks but died of multiorgan failure. Ghannam *et al*. [5] reported this approach using the CentriMag system as a bridge to LVAD. To the best of our knowledge, this is the first report describing technical modifications to make this approach safer. We improved the mismatch between the diameters of the cannula and the CentriMag cuff, and the use of the Ethibond suture undoubtedly gives better fixation. Finally, securing the cannula with two Hollister devices limits the probability of displacement. Although peripheral ECMO and other percutaneous VADs remain the initial choice in cardiogenic shock, there are many advantages of off-pump apical PROTEK Duo-RD, which include the elimination of ischemic leg complications, very effective LV decompression, decreased clot formation, less vascular complications and intravascular hemolysis, no afterload issues, patients can be extubated and ambulate early without any groin cannulas. In case of RV failure, a single 21 Fr cannula in the internal jugular vein can be attached with the inflow limb of the Protek Duo on ‘Y’, creating a ‘hybrid’ central VA ECMO. Placement of an axillary Impella 5.5 likely would have been the most acceptable alternative strategy, but it was not available then. Although this approach looks promising and safe, further study is required to identify the best-case scenario for this technique.
